# Extreme Left Ventricular Hypertrophy in Pediatric Hypertrophic Obstructive Cardiomyopathy

**DOI:** 10.7759/cureus.106450

**Published:** 2026-04-05

**Authors:** Elma Smajlović, Alma Bolic Alic

**Affiliations:** 1 Department of Pediatrics, Cantonal Hospital Zenica, Zenica, BIH

**Keywords:** hypertrophic cardiomayopathy, implantable cardioverter-defibrillator, septal myectomy, sudden cardiac death (scd), ventricular tachycardia (vt)

## Abstract

Hypertrophic cardiomyopathy (HCM) in childhood is characterized by marked phenotypic heterogeneity and poses significant challenges in risk stratification for sudden cardiac death (SCD). We report the case of a 9.5-year-old girl in whom transthoracic echocardiography revealed progressive extreme interventricular septal hypertrophy (up to 33 mm; Z-score > +11), marked left atrial enlargement, reduced left ventricular cavity size, and significant left ventricular outflow tract obstruction. Although repeated ambulatory Holter monitoring did not reveal ventricular arrhythmias, the patient exhibited one episode of unexplained syncope at rest. These findings placed her in a high-risk category according to contemporary pediatric risk stratification approaches. Following insufficient response to medical therapy, surgical septal myectomy was performed. Given the persistent high-risk profile, an implantable cardioverter-defibrillator was implanted for primary prevention of sudden cardiac death. This case emphasizes the importance of individualized, multimodal risk stratification in guiding management decisions in pediatric HCM, even in the absence of documented malignant arrhythmias.

## Introduction

Hypertrophic cardiomyopathy (HCM) in childhood represents a heterogeneous group of genetically determined myocardial disorders characterized by left ventricular or biventricular hypertrophy. It is among the most common inherited cardiomyopathies in the pediatric population [1]. Childhood-onset HCM, occurring between 1 and 18 years of age, constitutes a distinct clinical entity with regard to its underlying etiologies, modes of presentation, and patterns of disease progression [2]. In childhood-onset HCM, mutations in sarcomeric genes encoding contractile proteins account for approximately 50-75% of cases, with more than 1,400 pathogenic variants described, most commonly involving the *MYH7* and *MYBPC3* genes [1]. The disease is typically inherited in an autosomal dominant manner and is characterized by marked genotype-phenotype variability, incomplete penetrance, and heterogeneous clinical expression [1]. From a hemodynamic and morphological perspective, HCM is broadly divided into obstructive and non-obstructive forms, depending on the presence of dynamic left ventricular outflow tract obstruction, which significantly impacts symptom burden, disease progression, and risk stratification. Heterogeneous myocardial remodeling and dynamic changes in ventricular mechanics contribute to variability in clinical presentation. Patients may remain asymptomatic or, as hypertrophy progresses and left ventricular outflow tract (LVOT) obstruction develops, experience syncope, dyspnea, heart failure, and an increased risk of malignant arrhythmias and sudden cardiac death (SCD) [2,3]. In children with hypertrophic obstructive cardiomyopathy (HOCM), one of the major clinical challenges remains accurate risk stratification and identification of individuals at increased risk for adverse events, including ventricular tachycardia (VT), ventricular fibrillation (VF) and SCD [3]. Risk stratification in pediatric HCM differs fundamentally from adult populations, as disease expression and prognostic markers evolve with growth and development [3]. Disease outcomes are further shaped by heterogeneous responses to therapy, influenced by age-dependent factors such as metabolic rate, drug metabolism, and somatic growth and development. Based on contemporary pediatric risk stratification models, our patient demonstrates an intermediate-to-high estimated risk of sudden cardiac death, which is why we emphasize the importance of individualized risk assessment and discuss contemporary diagnostic and therapeutic approaches in pediatric HOCM.

## Case presentation

**Figure 1:**
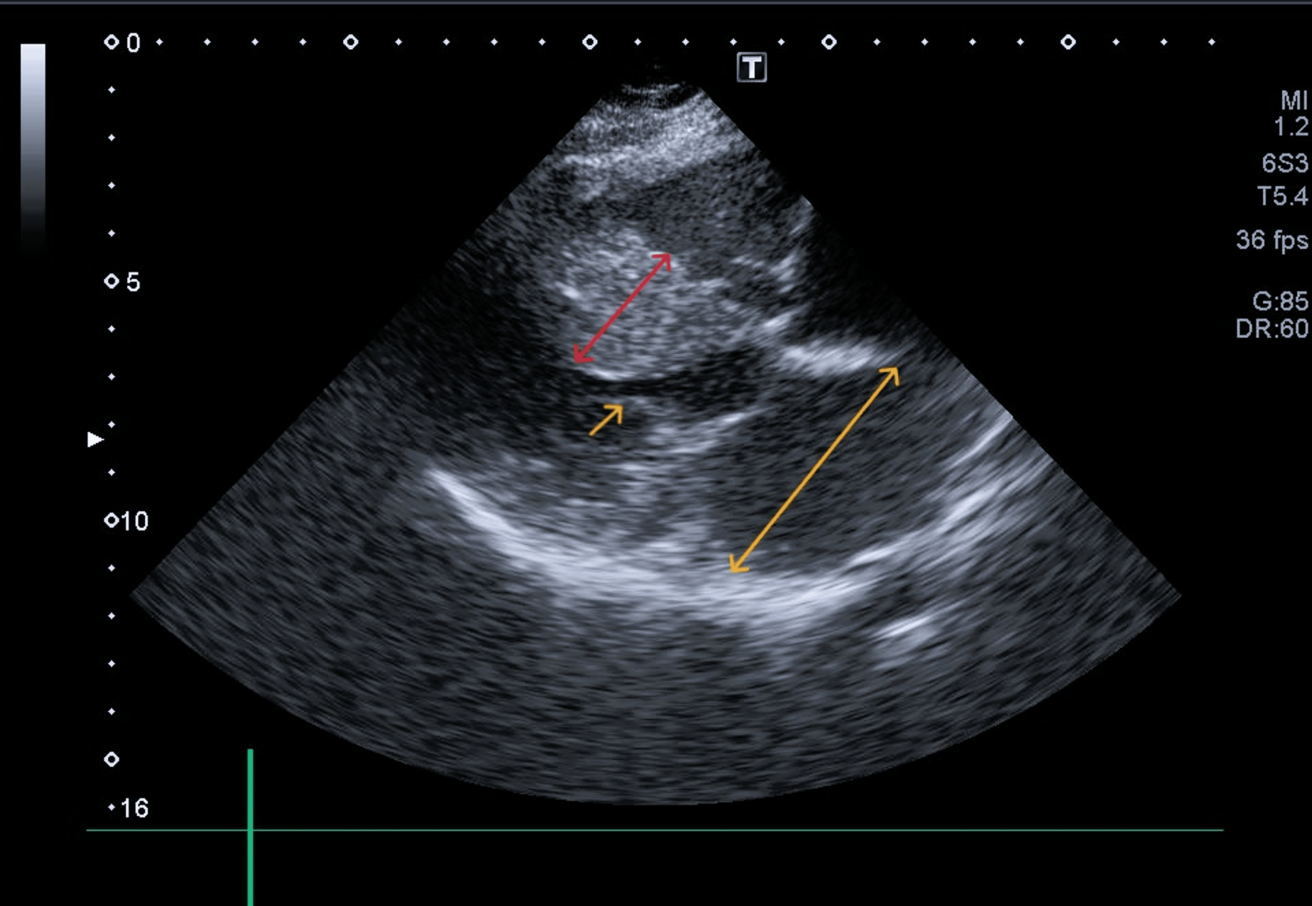
Extreme asymmetrical septal hypertrophy with systolic anterior motion of the anterior mitral valve leaflet Parasternal long-axis transthoracic echocardiographic view demonstrating marked asymmetric hypertrophy of the interventricular septum (red caliper), left atrial enlargement (yellow caliper), and systolic anterior motion of the anterior mitral valve leaflet (yellow arrow), contributing to left ventricular outflow tract obstruction.

**Figure 2:**
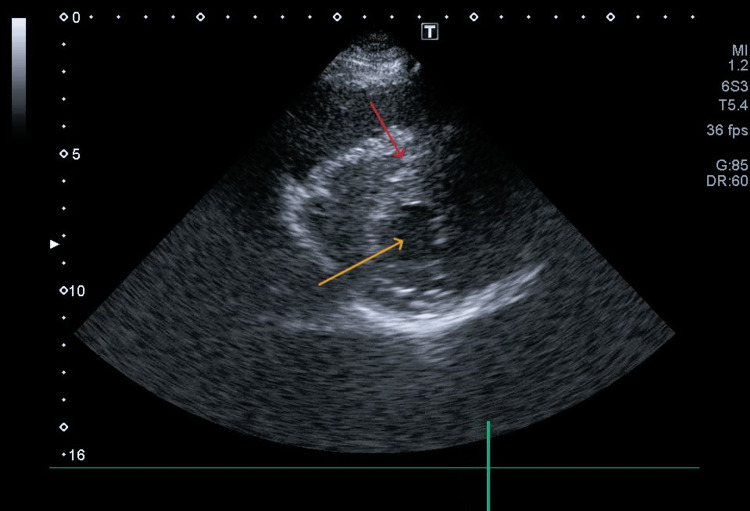
Marked reduction of the left ventricular cavity due to septal hypertrophy Parasternal short-axis transthoracic echocardiography view demonstrating extreme hypertrophy of the interventricular septum (red arrow), with marked reduction of the left ventricular cavity (yellow arrow).

**Figure 3:**
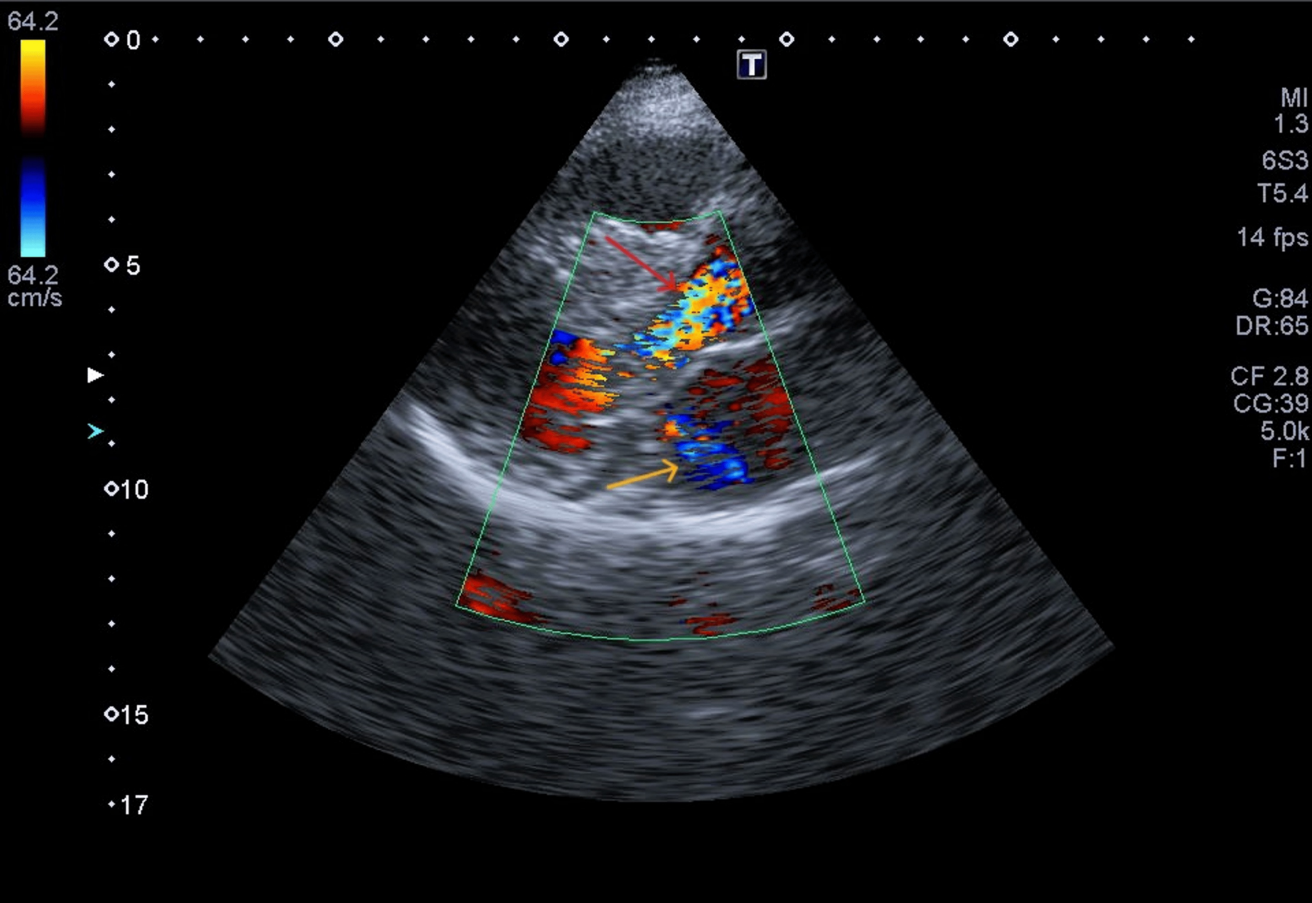
Dynamic left ventricular outflow tract obstruction and mitral regurgitation Parasternal long-axis color Doppler transthoracic echocardiographic view demonstrating turbulent systolic flow across the left ventricular outflow tract consistent with dynamic obstruction (red arrow), along with a posteriorly directed mitral regurgitation jet (yellow arrow).

A 9.5-year-old girl was admitted to our department due to hemorrhagic enteritis, during which a grade III/6 holosystolic murmur, best heard along the left sternal border, was detected for the first time. The remainder of the physical examination, including detailed cardiac and peripheral vascular examination, as well as laboratory investigations, was unremarkable. Subsequent history revealed exertional fatigue, with occasional headaches and dizziness. Family history revealed that the patient's mother had a diagnosis of non-obstructive hypertrophic cardiomyopathy, while her twin sister's echocardiographic evaluation was normal at the time. Transthoracic echocardiography (TTE) demonstrated extreme interventricular septal hypertrophy (IVSD 27 mm, Z-score +8.5), reduced left ventricular end-diastolic dimension (LVEDD 33 mm, Z-score −3.5), and left atrial dilatation (LA 39 mm, Z-score +3.9), accompanied by systolic anterior motion of the anterior mitral leaflet (SAM) and left ventricular outflow tract (LVOT) obstruction with a peak gradient 20 mmHg (Figures 1-3).

**Figure 4:**
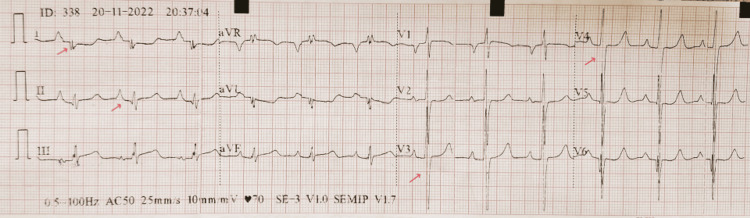
Electrocardiographic findings in hypertrophic obstructive cardiomyopathy Electrocardiography findings included right axis deviation, first-degree AV block, tall P waves (3mm), deep S waves in leads V2-V4, and nonspecific interventricular conduction abnormalities (red arrows).

Electrocardiography (ECG) revealed right axis deviation, first-degree atrioventricular block, tall P waves (3 mm), deep S waves in leads V2-V4, and nonspecific intraventricular conduction abnormalities (Figure 4).

**Figure 5:**
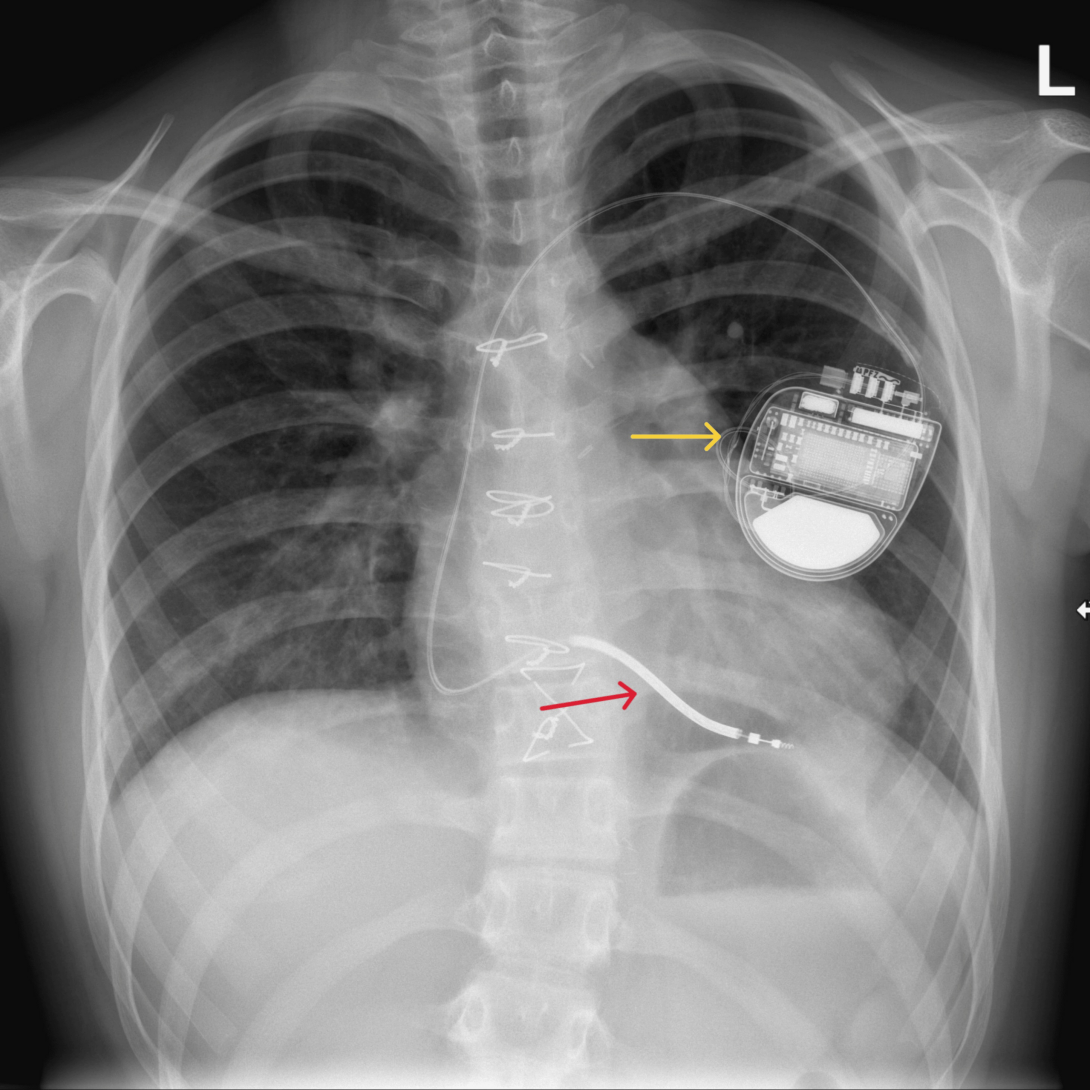
Chest X-ray demonstrating a left-sided single-chamber ICD Chest radiograph demonstrating a left-sided transvenous implantable cardioverter-defibrillator (ICD) with the pulse generator positioned in the left infraclavicular region (yellow arrow) and a single transvenous lead, with the distal tip positioned in the right ventricular apex and the defibrillation coil visible (red arrow).

The diagnosis of hypertrophic obstructive cardiomyopathy was established based on echocardiographic findings of severe asymmetric left ventricular hypertrophy accompanied by dynamic left ventricular outflow tract obstruction, while electrocardiographic abnormalities provided additional supportive evidence. Genetic testing identified a heterozygous variant of uncertain significance (VUS) in the *MYH7* gene, which was inherited from the patient's mother. The patient was started on beta-blocker therapy (1 mg/kg propranolol) with restriction of participation in competitive sports and scheduled follow-up in six months. During the following year, two 24-hour Holter ECG recordings showed no arrhythmias. However, echocardiographic follow-up demonstrated progression of septal hypertrophy (IVSD 33 mm, Z-score +11.1), further left atrial enlargement (LA 45 mm, Z-score +6.1), development of moderate mitral regurgitation, and worsening LVOT obstruction with a peak gradient of 50 mmHg. Interestingly, the patient reported a subjective improvement in exercise tolerance. Despite good adherence to medical therapy and consistent follow-up, the obstruction remained hemodynamically significant, requiring surgical management. After cardiothoracic surgical consultation, septal myectomy was indicated and successfully performed without complications. Postoperative TTE showed a reduction of LVOT gradient to 20 mmHg. In the same year, the patient experienced a transient loss of consciousness occurring at rest (while seated in a car), suggestive of syncope. In accordance with current European Society of Cardiology (ESC) recommendations, implantable cardioverter-defibrillator (ICD) implantation was indicated, given the presence of major risk factors for SCD, particularly unexplained syncope and extreme left ventricular hypertrophy. Implantation of a transvenous ICD was recommended and performed at another institution abroad (Figure 5), and medical therapy was switched to metoprolol (approximately 1,8 mg/kg/day), presumably to optimize beta-blockade and tolerability.

Over the subsequent three-year follow-up, the patient remained clinically stable, without recurrent syncope. Echocardiographic findings remained unchanged (IVSD 33 mm, LA 45 mm, MR grade 2+, LVOT peak gradient 20 mmHg), as did the surface ECG. Repeated 24-hour ECG Holter recordings revealed only isolated unifocal ventricular premature beats. ICD interrogation showed no ventricular arrhythmias and no delivered or aborted therapies. At the most recent ICD evaluation, one short episode of non-sustained ventricular tachycardia (six consecutive ventricular ectopic beats) was recorded, remaining below the programmed detection threshold; therefore, no ICD therapy or aborted shock was delivered. The patient also reported a single episode of transient loss of consciousness for which medical attention was not sought. At the latest follow-up visit, malposition of the ICD generator toward the left axilla with local pressure ulceration was noted, prompting surgical revision and repositioning of the device. On follow-up screening, the twin sister was found to have newly developed signs of hypertrophic cardiomyopathy, with interventricular septal hypertrophy (IVSD 15 mm) but without LVOT obstruction. Continued clinical and echocardiographic surveillance of both sisters is planned.

## Discussion

HCM in childhood represents a clinically heterogeneous condition with a broad spectrum of phenotypic expression and outcomes. Diagnosis is primarily established by transthoracic echocardiography, with left ventricular wall thickness exceeding two standard deviations above the predicted mean (Z-score > +2) considered diagnostic [3]. Wall thickness below this threshold, even in the presence of a positive family history, may raise suspicion but is not sufficient for diagnosis [3]. In the present case, interventricular septal thickness exceeded 30 mm, corresponding to a Z-score greater than +11 according to Boston nomograms [4], consistent with extreme myocardial hypertrophy. This was accompanied by significant LVOT obstruction, reduced LV cavity size, and progressive LA enlargement, reflecting advanced structural disease. Extreme hypertrophy has consistently been identified as one of the strongest morphological predictors of adverse outcomes in pediatric HCM and is associated with an increased risk of arrhythmic events and SCD [5,6]. Genetic analysis identified a maternally inherited heterozygous variant of uncertain significance in the *MYH7* gene, a key sarcomeric gene associated with autosomal dominant hypertrophic cardiomyopathy. 

The presence of a first-degree relative with milder disease expression is consistent with the autosomal dominant inheritance of HCM, characterized by incomplete penetrance and variable expressivity [7]. However, given its classification as a variant of uncertain significance, a definitive genotype-phenotype correlation cannot be established. Clinical presentation in pediatric HCM ranges from asymptomatic disease to exertional dyspnea, palpitations, syncope, and SCD. In this patient, initial symptoms were mild; however, the subsequent occurrence of unexplained syncope at rest represented a major clinical risk marker [6]. The primary long-term therapeutic goals in pediatric HOCM are reduction of LVOT obstruction, symptom control, prevention of malignant ventricular arrhythmias, and identification of patients at increased risk of SCD. Beta-blockers remain the first-line pharmacological therapy in symptomatic patients, aiming to reduce heart rate, improve diastolic filling, and attenuate dynamic obstruction. Although high-quality comparative studies are lacking and no specific beta-blocker is universally recommended, small studies, particularly with metoprolol, have demonstrated beneficial effects on symptom control and hemodynamic parameters [8].

When optimal medical therapy fails to reduce the LVOT gradient below 50 mmHg, septal myectomy remains the gold standard treatment in pediatric patients [9]. Surgical intervention has been shown to effectively reduce postoperative LVOT gradients, alleviate SAM, and decrease the severity of mitral regurgitation. In our patient, persistent severe obstruction despite beta-blocker therapy warranted surgical septal myectomy. Despite successful surgical intervention, certain patients remain at increased risk for SCD. Risk stratification models developed for adult populations cannot be directly extrapolated to children, leading to the development of pediatric-specific approaches. Contemporary studies consistently identify extreme left ventricular wall thickness, left atrial enlargement, unexplained syncope, and age as the most relevant risk factors, whereas left ventricular outflow tract gradient, family history, and genetic findings show less consistent prognostic value [6,10]. Previous studies have demonstrated that ECG-based risk scores, incorporating QRS voltage and repolarization abnormalities, may enhance sudden cardiac death risk stratification in childhood hypertrophic cardiomyopathy; however, such tools should be interpreted in conjunction with echocardiographic and clinical risk factors [11].

While not demonstrating the most predictive ECG features, the observed findings in our patient would still contribute to ECG-based risk scores, indicating underlying electrical remodeling. According to contemporary pediatric risk stratification approaches, extreme myocardial hypertrophy and unexplained syncope represent major independent risk factors for sudden cardiac death, supporting the decision for primary prevention with ICD implantation. ICD therapy in children is associated with unique challenges related to patient size, growth, and device-related complications; however, in carefully selected high-risk patients, the potential benefit outweighs these limitations. In children, ICDs are most commonly implanted epicardially in younger patients, while transvenous and subcutaneous systems are more frequently used in adolescents [12]. Newer-generation devices, including the subcutaneous ICD (S-ICD), have improved the overall benefit-to-risk ratio in high-risk pediatric populations [12]. Although concerns remain that current pediatric risk models may overestimate the true incidence of adverse events, periodic reassessment of risk and close monitoring of high-risk patients remain essential components of long-term management [10]. 

## Conclusions

This case highlights the importance of comprehensive risk stratification in pediatric hypertrophic cardiomyopathy, integrating clinical presentation, imaging findings, electrocardiographic abnormalities, and family history. It also underscores the rarity and clinical significance of extreme myocardial hypertrophy in childhood, which represents a major risk factor for adverse outcomes. Furthermore, it illustrates the variability of phenotypic expression despite shared genetic background, as well as the limitations of genetic testing in the presence of variants of uncertain significance. Early recognition of high-risk features is essential to guide timely therapeutic decisions, including surgical intervention and consideration of implantable cardioverter-defibrillator therapy.

## References

[1] Norrish G, Field E, Kaski JP (2021). Childhood hypertrophic cardiomyopathy: a disease of the cardiac sarcomere. Front Pediatr.

[2] Marston NA, Han L, Olivotto I (2021). Clinical characteristics and outcomes in childhood-onset hypertrophic cardiomyopathy. Eur Heart J.

[3] Arbelo E, Protonotarios A, Gimeno JR (2023). 2023 ESC Guidelines for the management of cardiomyopathies. Eur Heart J.

[4] Colan SD (2013). The why and how of Z scores. J Am Soc Echocardiogr.

[5] Norrish G, Ding T, Field E (2022). Relationship between maximal left ventricular wall thickness and sudden cardiac death in childhood onset hypertrophic cardiomyopathy. Circ Arrhythm Electrophysiol.

[6] Norrish G, Ding T, Field E (2019). Development of a novel risk prediction model for sudden cardiac death in childhood hypertrophic cardiomyopathy (HCM risk-kids). JAMA Cardiol.

[7] Mahmood A, Khalid H, Mushtaq A (2025). Genetic and clinical perspectives in hypertrophic cardiomyopathy: a systematic review and meta- analysis on risk stratification for sudden cardiac death. Cardiolo Cardiovas Med.

[8] Dybro AM, Rasmussen TB, Nielsen RR, Andersen MJ, Jensen MK, Poulsen SH (2021). Randomized trial of metoprolol in patients with obstructive hypertrophic cardiomyopathy. J Am Coll Cardiol.

[9] Cappellaro AP, de Almeida LF, Gismondi RA, Ayala R, Thet MS, Dearani JA (2025). Septal myectomy in pediatric obstructive hypertrophic cardiomyopathy: a systematic review and meta-analysis. Pediatr Cardiol.

[10] Wilkin M, Khraiche D, Panaioli E (2025). Independent external evaluation of pediatric hypertrophic cardiomyopathy risk scores in predicting severe ventricular arrhythmias. Circ Arrhythm Electrophysiol.

[11] Östman-Smith I, Sjöberg G, Rydberg A, Larsson P, Fernlund E (2017). Predictors of risk for sudden death in childhood hypertrophic cardiomyopathy: the importance of the ECG risk score. Open Heart.

[12] Silvetti MS, Bruyndonckx L, Maltret A (2023). The SIDECAR project: S-IcD registry in European paediatriC and young Adult patients with congenital heaRt defects. Europace.

